# An Updated Comprehensive Review on Vitamin A and Carotenoids in Breast Cancer: Mechanisms, Genetics, Assessment, Current Evidence, and Future Clinical Implications

**DOI:** 10.3390/nu13093162

**Published:** 2021-09-10

**Authors:** Jee Ah Kim, Ja-Hyun Jang, Soo-Youn Lee

**Affiliations:** 1 Department of Laboratory Medicine and Genetics, Samsung Medical Center, Sungkyunkwan University School of Medicine, 81 Irwon-ro, Gangnam-gu, Seoul 06351, Korea; aziron89@gmail.com (J.A.K.); jahyun.jang@samsung.com (J.-H.J.); 2 Department of Clinical Pharmacology & Therapeutics, Samsung Medical Center, Sungkyunkwan University School of Medicine, 81 Irwon-ro, Gangnam-gu, Seoul 06351, Korea; 3 Department of Health Science and Technology, Samsung Advanced Institute of Health Science and Technology, Sungkyunkwan University, 115 Irwon-ro, Gangnam-gu, Seoul 06355, Korea

**Keywords:** vitamin A, retinoids, carotenoids, breast cancer, cancer risk, cancer signaling, prevention, treatment, novel targets

## Abstract

Vitamin A and carotenoids are fat-soluble micronutrients that play important role as powerful antioxidants modulating oxidative stress and cancer development. Breast cancer is the most common malignancy in women. As the risk of breast cancer is dependent on various lifestyle factors such as dietary modifications, there is increasing interest surrounding the anti-cancerous properties of vitamin A and carotenoids. Despite the suggested protective roles of vitamin A and carotenoids in breast cancer development, their clinical application for the prevention and treatment of breast cancer is limited. In this narrative review, we discuss the roles of vitamin A and carotenoids along with the evaluation method of vitamin A status. We also exhibit the association of genetic variations involved in metabolism of vitamin A and carotenoids with cancers and other diseases. We demonstrate the epidemiological evidence for the relationship of vitamin A and carotenoids with breast cancer risk, their effects on cancer mechanism, and the recent updates in clinical practice of vitamin A or carotenoids as a potential therapeutic agent against breast cancer. This review provides insight into the preventive and therapeutic roles of vitamin A and carotenoids in breast cancer development and progression.

## 1. Introduction

**Figure 1:**
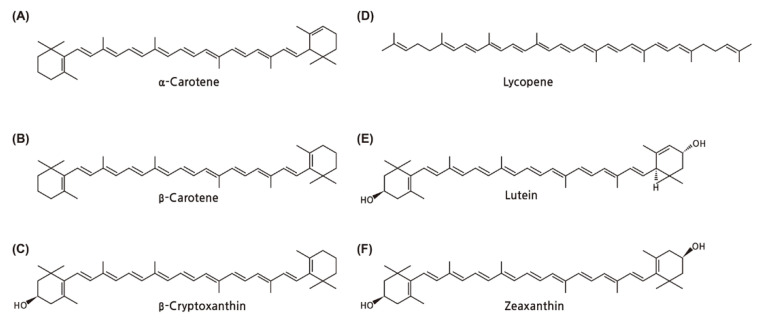
(**A**) α-carotene, (**B**) β-carotene, and (**C**) β-cryptoxanthin are classified as provitamin A, which can be converted into retinol in the human body. The other carotenoids, including (**D**) lycopene, (**E**) lutein, and (**F**) zeaxanthin, are non-provitamin A, which cannot be converted into retinol.

Vitamin A is the nutritional term for the group of fat-soluble unsaturated hydrocarbons. It includes retinol and its derivatives (retinal, retinoic acid, and retinyl ester), which are collectively called retinoids. Carotenoids are organic pigments that are responsible for yellow, orange, and red colors and are mainly found in fruit and vegetables. Carotenoids can be classified into two groups: some carotenoids (α-carotene, β-carotene, and β-cryptoxanthin) are called provitamin A, as they can be metabolized into retinol in the intestine and become a natural source of retinoids (Figure 1). The other carotenoids (lycopene, lutein, and zeaxanthin) are non-provitamin A as they cannot be converted into retinol, but are still suggested to have some biological effects as antioxidants aside from vitamin A activity [1]. The vitamin should be obtained via preformed vitamin A from animal products or as provitamin A carotenoids in fruits and vegetables. Vitamin A has generally been accepted to be associated with a number of beneficial biological activities, such as protecting from photo-energy, enhancing the immune system, and modulating oxidative stress [2]. Vitamin A and carotenoids are reported to regulate the proliferation, growth, and differentiation of tumor cells. Through diverse epidemiological studies, the preventive effects of vitamin A and carotenoids have been revealed in numerous chronic health diseases as well as tumorigenesis.

According to the World Health Organization (WHO), cancer is the second most attributable cause of death globally. Among malignant neoplasms, breast cancer is the most common malignancy occurring in women, with 2.26 million newly diagnosed cases in 2020 [3,4,5]. As the overall survival rates of breast cancer increase, the importance of environmental factors, such as dietary patterns and nutrient intake, has become more emphasized [6,7,8]. Vitamins are essential micronutrients involved in diverse physiological mechanisms, acting as antioxidants [9]. Recently, there has been a growing interest in vitamin A and carotenoids as anti-cancerous compounds against several cancers. Numerous epidemiological studies have suggested low intake and serum concentration of vitamin A and carotenoids as risk factors for breast cancer [10,11,12]. However, the exact functional roles and mechanisms of vitamin A and carotenoids in breast cancer development and progression are still not fully identified due to the inconclusive results in the relevant literature. The data’s inconsistency may have resulted from different analytes for vitamin A assessment alongside biological variation as vitamin A status can be easily affected by its absorption, metabolism, and bioavailability [13,14]. The current review describes the biological functions of vitamin A and carotenoids, the assessment of vitamin A status, and the association of genetic variations involved in metabolism of vitamin A or carotenoids with cancers and other diseases. We explore the link of vitamin A and carotenoids with breast cancer through epidemiological investigations and discuss the recent updates and advances in the potential preventive and therapeutic roles of vitamin A and carotenoids in breast cancer.

## 2. Structure, Sources, and Absorption of Vitamin A and Carotenoids

**Figure 2:**
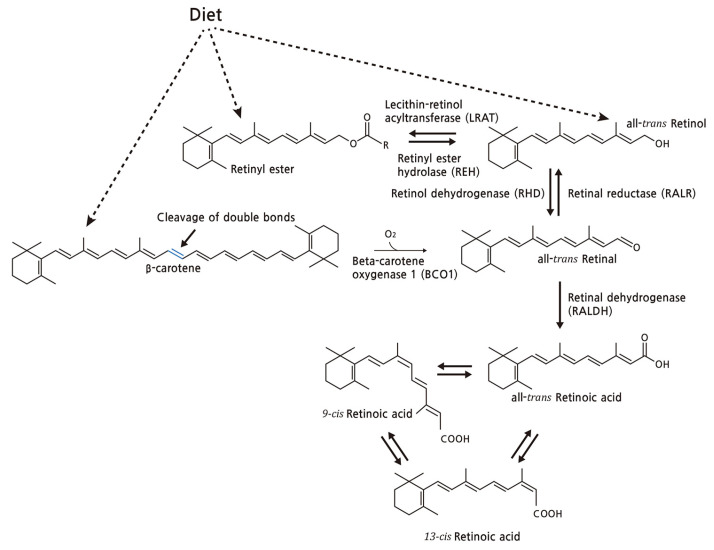
The metabolic conversion of β-carotene and retinoids. From the diet, β-carotene is taken up by the enterocyte and is metabolized into all-*trans*-retinal via beta-carotene oxygenase 1 (BCO1). Similarly, retinyl ester or retinol obtained from the diet is converted to all-*trans*-retinal via retinyl ester hydrolase (REH) and retinal reductase (RALR), and becomes further oxidized into all-*trans*-retinoic acid (ATRA), which is responsible for the genetic regulation of vitamin A, via retinal dehydrogenase (RALDH) in the target cell. Through the non-enzymatic process, ATRA can be isomerized to 9-*cis*-retinoic acid and 13-*cis*-retinoic acid, and vice versa [17].

Vitamin A, also known as retinoids, is a 20-carbon molecule comprised of a cyclohexenyl ring with methyl-substitution (β-ionone ring) and a conjugated polyene chain (–C=C–) structure (isoprenoid side chain) with a different functional group at the terminal C15 [9,14,15,16]. Carotenoids are tetraterpenoids in which two 20-carbon structures containing β-ionone rings and a polyisoprenoid side chain are linked together by tail-to-tail. Based on their chemical structure, carotenoids can be divided into carotenes (e.g., α-carotene, β-carotene, β-cryptoxanthin, and lycopene), which refer to hydrocarbons in general, and xanthophylls (e.g., lutein and zeaxanthin), which contain oxygen atoms in the form of hydroxyl group. From the diet, β-carotene is metabolized into retinal in the intestine and becomes a natural source of retinoids (Figure 2). Similarly, other retinoids, such as retinyl ester and retinol obtained directly from nutritional intake, are converted into retinal. Retinoids share similar molecular structures and functions but with variable potency in absorption and interconversions from one form to another. In the form of all-*trans*-retinoic acid (ATRA), 13-*cis*-retinoic acid, and 9-*cis*-retinoic acid, these isomers are approved to be transcriptionally active [17,18].

As the human body cannot produce vitamin A, it needs to be acquired from the diet in either preformed vitamin A or the form of provitamin A carotenoids. Unlike preformed dietary vitamin A, which is well absorbed (about 70–90%) in the human body, provitamin A carotenoids can only be absorbed 3% or less [19,20]. In addition, these substances are necessary to be converted into a form of vitamin A through a series of reactions in human intestinal cells. As the conversion of carotenoids into vitamin A is affected by various factors, provitamin A carotenoids are highly variable and less reliable source of the vitamin than preformed vitamin A from animal products [21]. The conversion rate depends on the food matrix, food preparation methods, and the amount and type of fat in the diet. Foods with a simpler matrix, such as fruit and red palm oil, tend to have high bioavailability of carotenoids. Thermal processing has been reported to increase the bioavailability and absorption of β-carotene [22]. Therefore, cooked vegetables have enhanced bioavailability than raw vegetables. In addition, dietary fat such as olive oil is essential for carotenoids to be absorbed in the intestine and promotes carotenoids to incorporate with micelles [23,24,25]. On the other hand, dietary fibers or deficiencies of some micronutrients, including zinc and iron, reduce the ability of carotenoids to convert into vitamin A. Although more than 600 natural structural variants of carotenoids exist, only ~40 carotenoids are consumed in the human diet, and approximately 20 carotenoids have been found in human blood and tissues [26]. Among six carotenoids, including β-carotene, α-carotene, lycopene, lutein, zeaxanthin, and β-cryptoxanthin, that make up more than 95% of the total carotenoids in blood, β-carotene constitutes the most significant provitamin A activity, with other carotenoids possessing approximately half of the vitamin A activity of β-carotene [19]. However, the absorption of β-carotene from most plant foods has been estimated to be 7–65%, indicating the limitations of the carotenoids activity in humans. Carotenoid content is severely restricted in the amount contained in food, amount of absorbance, food source, and the production of enzymes that facilitate the conversion of carotenoid to vitamin A. The degree of ripeness of fruits or vegetables, technique and location of cultivation, and the method of drying before storage tend to significantly affect the content of carotenoids. Carotenoids are taken up into the enterocyte through the cluster of differentiation 36 (CD36) transporter or the scavenger receptor B1 (SRB1) by passive diffusion [18,27]. Carotenoids are transformed into retinyl esters or β-carotene through a series of reactions and are secreted into the bloodstream associated with micelles. The absorption and metabolism of vitamin A and carotenoids in the intestine and the role of the proteins involved are described in detail in these recent updates from Carazo et al. [28].

## 3. Antioxidant Properties and Other Functions of Vitamin A and Carotenoids

Production of reactive oxygen and nitrogen species during aerobic metabolism is known to be associated with degenerative processes [29]. Vitamin A and carotenoids have been found to be associated with several pathological status such as cardiovascular diseases, diabetes, osteoporosis, skin diseases, and cancers [13]. Recent studies have focused on the protective effects of vitamin A and carotenoids as antioxidants. In particular, carotenoids can act as very efficient quenchers of singlet oxygen, both in vitro and in vivo. They react with other free radicals, breaking them down into biologically active degradation products [30]. Conjugated double bonds in carotenoids absorb electrons from singlet oxygen (^1^O_2_), thus neutralizing reactive oxygen species (ROS) and reactive nitrogen species [15,26]. Therefore, the length of the polyene chain significantly affects the functional antioxidant activity of carotenoids [14]. Carotenoids quench free radicals through several mechanisms involving electron transfer, radical adduct formation, and hydrogen atom transfer [13,15]. Lycopene is reported to efficiently reduce reactive oxygen produced by smoke, and zeaxanthin is able to quench both water- and fat-soluble peroxyl radicals [26]. In addition, the ability to scavenge reactive nitrogen species is enhanced when two fat-soluble antioxidants such as β-carotene or vitamin E are combined [29].

The association of vitamin A and carotenoids in the development of several cancers, including breast, cervix, skin, prostate, oral cancer, and leukemia, has been highlighted in many studies [10]. Retinoids affect the growth of malignant cells through growth arrest, apoptosis, and re-differentiation [31,32]. A study reported that vitamin A deficiency due to poor diet may be one of the contributing factors in cancer development [33]. Contrarily, high dose supplementation of carotenoids increased the risk of lung cancer in smokers [34,35]. In the case of the roles of vitamin A and carotenoids in breast cancer, the conversion of retinol into retinoic acid was found to be impaired in breast cancer cell lines [36]. In addition, treatment using carotenoids in human breast cancer cell lines was reported to inhibit cell proliferation and increase apoptosis [37]. Several epidemiological studies demonstrated that the serum retinol or β-carotene level was significantly decreased in those with breast cancer [38,39,40]. Furthermore, breast cancer patients with progressed tumor stages tend to have reduced serum concentrations of vitamin A and carotenoids [41,42,43,44]. The concentrations of vitamin A and carotenoids were inversely associated with breast cancer risk [45,46,47,48,49,50,51,52], suggesting the protective potency of vitamin A and carotenoids against breast cancer development. However, there are conflicting results that vitamin A and carotenoids do not have significant protective effects against breast cancer [53,54,55,56,57,58].

Several active forms of vitamin A, such as ATRA, 9-*cis*-retinoic acid, and 13-*cis*-retinoic acid, have major biological functions in human body. Vitamin A is essential for vision, cell growth, tissue differentiation, and the immune system. The visual system is mediated by chromophores, which are complexes of 11-*cis*-retinal bound to a protein, called an opsin. When a photon is absorbed, 11-*cis*-retinal photochemically isomerizes into all-*trans*-retinal, which causes the change of the opsin protein leading to signal transduction. This allows the photoreceptor cells to change the rate of glutamate release. By regenerating 11-*cis*-retinal from all-*trans*-retinal in the eye, the vision can proceed [59,60]. In addition, vitamin A is essential for numerous parts of embryo development. Retinoic acid acts in neural differentiation and the development of major organs, including the heart, kidney, respiratory tract, eyes, urinary tract, and the skeleton. Recently, there have been study results indicating that vitamin A is required for the formation of the reproduction system in both males and females [61]. Vitamin A is also required for both innate and adaptive immunity. It not only affects the activation of neutrophils and macrophages, but also regulates the differentiation of T-helper cells and B cells [62,63,64].

## 4. Vitamin A Status Assessment

Stored vitamin A is released into the bloodstream as retinol when necessary [65,66]. Retinol circulates in the plasma in a complex bound to retinol binding protein (RBP) and transthyretin (TTR). Therefore, vitamin A status can be influenced by the patient’s physiological state, which can affect the protein balance—such as through protein malnutrition, liver disease, acute inflammation or infection, and aberrant levels of C-reactive protein [9,67]. Gastrointestinal disorders, such as Crohn’s disease, celiac disease, or pancreatic disorders or particular nutritional deficiencies, including iron and zinc, can affect vitamin A absorption or metabolism [68]. Serum or plasma concentrations of retinol or carotenoids in fasting states are often measured to determine vitamin A status. However, these concentrations do not directly represent the exact status of vitamin A, as the blood level maintains homeostasis unless vitamin A stored in the liver becomes critically depleted or overloaded [69].

There is growing evidence that the measurement of circulating retinol or carotenoid concentrations may be a more potential biomarker for antioxidant status than dietary data [70,71]. Assess of dietary intakes based on food frequency questionnaires has several weaknesses such as inaccuracies through recall of past diet, variations in the amount of nutrient absorption in each individual, and difficulties in reflecting the exact amount of carotenoid content in specific foods that have been modified through different forms of cooking and storage [72,73,74,75]. Due to these limitations, blood concentrations of retinol and carotenoids have been found to have a negligible to moderate correlation with fruit and vegetable intake (*r* = 0.04–0.06 for retinol and *r* = 0.2–0.7 for carotenoids) [76,77,78,79].

Accurate determination of the blood concentration of vitamin A is critical, as toxic or deficient levels of vitamin A have been associated with various diseases. Previously, the determination of vitamin A level was assessed by directly measuring a blue pigment produced when vitamin A reacts with antimony trichloride (Carr–Price reaction) or trifluoroacetic acid (Neeld–Pearson reaction). These methods were time-consuming, not easily automated, and could be influenced by interferences [80]. High-pressure liquid chromatography (HPLC) with fluorometric or spectrophotometric detection has since been widely used as the primary standard method. The absorbance of vitamin A or carotenoids is photometrically detected at wavelengths of 325 or 450 nm, and quantification is made by peak-height-ratios normalized with the internal standards [81,82]. HPLC methods have been proved to improve in terms of both sensitivity and specificity compared to the previous photometric methods. However, measuring fat-soluble vitamin A and carotenoids with HPLC methods has several difficulties; HPLC approaches require extraction and concentration phases to remove the oily matrix and enrich the target analyte in order to detect fat-soluble vitamins. In general, liquid–liquid extraction, solid-phase extraction, and supported-liquid extraction have been widely used for sample pretreatment [9,83]. Analyzing vitamin A and carotenoids with HPLC is also hard as each analyte ionizes with great variety. Furthermore, as the concentration of the target analyte is extremely low, a large volume of sample and a timely run-time is required to measure the concentration [84].

Liquid chromatography coupled with tandem mass spectrometry (LC-MS/MS) is a recently developed method that has been shown to be superior to other widely used methods and thus became the most selective analytical procedure [84,85,86]. By using LC-MS/MS technology, many fat-soluble vitamins can now be quickly measured concurrently in a small amount of sample. This method can also isolate and quantify the isotypes or epimers of vitamins [85,87] and measure multiple components and many compounds within samples [88]. Various specimens with complex matrix samples can be examined, expanding the research area of vitamin A [89,90]. More accurate evaluation of vitamin A status has now become available due to the advanced technology of LC-MS/MS that has simplified the extraction step, reduced sample volume size, and shortened detection time [91].

It is well known that vitamin A samples are photosensitive. Albahrani et al. compared the stability of vitamin A in whole blood, serum, and extract by light, temperature, and time [92]. The study presented that vitamin A can tolerate light exposure for up to 48 h. However, light protection, such as wrapping in foil and storage at −20 °C, is recommended for collected samples if lab analysis is delayed for more than 48 h.

Vitamin A status is estimated by measuring serum or plasma retinol level using a reference interval (RI), which is commonly defined as the central distribution of values that are seen in a certain percentage (usually 95%) of healthy individuals [93,94]. Serum concentration for carotenoid deficiency has not yet been established [68]. When measuring serum vitamin A level with the LC-MS/MS method, guidance RI for adults is 0.3–0.8 mg/L [9,95]. Yin et al. proposed RIs for serum vitamin A levels in the elderly (aged 64–88 years) as 0.283–0.730 mg/L, with no difference between sex [93]. Values above 0.3 mg/L represent the significant storage of vitamin A in the liver and correlate well with vitamin A intake. However, plasma or serum concentration for vitamin A can be various depending on age, sex, inflammation, infection, seasonal variation, population, and methodology [67,96,97,98,99]. Therefore, it may be important to establish the appropriate RIs to accurately determine the individual’s vitamin status.

## 5. Genetic Variations in Genes Involved in Vitamin A and Carotenoids Metabolism and Their Association with Either Vitamin A Concentration or Diseases

Vitamin A interacts with various proteins in the process of metabolism, transportation, and biologic action. Therefore, variants in genes involved in vitamin metabolism could affect not only vitamin A concentration, but also human health.

In the genome-wide association study (GWAS) of 5006 Caucasian individuals, rs1667255 in *TTR* and rs10882272 in *RBP4* were found to be associated with serum retinol concentration, and the latter SNP was confirmed to be statistically significant in a replication study [100]. Approximately 95% of retinol in blood is found in RBP-bound form (RBP4), which in turn binds to TTR which stabilizes the complex. Two single nucleotide polymorphisms (SNPs), A379V (rs7501331), and R267S (rs12934922) in the coding region of *BCO1* are known to be associated with reduced activity of β-carotene 15,15′-monooxygenase (BCOM1) that catalyzes β-carotene into two molecules of retinal and increased level of fasting β-carotene concentration [101]. Another SNP located in the promoter region of *BCO1*, rs6564851, has been known to be associated with fasting β-carotene concentration by GWAS and candidate gene study [102,103]. The SNP was shown to affect the binding of intestine-specific homeobox (ISX) which acts as a repressor of β-carotene absorption and cleavage into retinal [104,105]. As those SNPs might be associated with the bioavailability of the β-carotene, it can be an essential issue for vegetarians in whom β-carotene is the primary source of vitamin A.

Pathogenic variants that impair the function of proteins can cause Mendelian diseases, and their phenotypes mainly involve the eyes. Mutations in the *RBP4* gene can cause either autosomal dominant microphthalmia (OMIM # 616428) or autosomal recessive retinal dystrophy, iris coloboma, and comedogenic acne syndrome (OMIM # 615147). Mutations in *STRA6*, of which protein is involved in retinol uptake in target cells, can cause Microphthalmia (OMIM # 601186) in an autosomal recessive manner. Bi-allelic mutations in *LRAT*, of which protein esterifies all-*trans*-retinol to all-*trans*-retinyl palmitate, can cause Leber congenital amaurosis 14 or retinal dystrophy 9 (OMIM # 613341). Mutations in RPE65, of which protein is involved in acyl cleavage and isomerization of all-*trans*-retinyl ester, can cause Leber congenital amaurosis 2 (OMIM # 204100) and retinitis pigmentosa 20 (OMIM # 613794). As *LRAT* and *RPE65* are involved in the generation of a chromophore, 11-*cis*-retinal, in retinal pigmented epithelial cells, mutations in those genes manifest as retinal diseases.

A polymorphism (rs2241057) in *CYP26B1*, involved in the degradation of retinoic acid, was suggested to be associated with Crohn’s Disease [106]. There are several association studies between the polymorphisms of vitamin A metabolism and cancer. In the study of neuroblastomas, an embryonic tumor originating from the neural crest, maternal polymorphisms rather than patients (offspring) were associated with the risk of tumors; rs12442054-proximal to *STRA6* was associated with the decreased risk of neuroblastomas [107]. In prostatic cancer, rs1330286 in *ALDH1A1* and rs4646653 in *ALDH1A3* were associated with cancer risk [108]. In a candidate gene approach (*TTR, FFAR4, BCO1, RARB, RARB, ABCA1*, and *FABP2*), there was no association in colorectal cancer patients [109]. In a study of *BCO1* polymorphisms and breast cancer risk, no significant association was observed [110]. Although studies exhibited inconsistent results, further research might be necessary for each cancer type, including enough samples to draw a conclusion regarding the association between polymorphisms in genes for vitamin A metabolism and cancer risk.

## 6. Associations of Vitamin A and Carotenoids with Breast Cancer Risk in Epidemiological Studies

We reviewed reported studies within the last two decades that investigated the blood concentrations of retinol and carotenoids with respect to the risk of breast cancer. Among them, prospective cohort studies or nested case–control studies comprised of a minimum of 150 breast cancer cases were included. All studies except one were assigned as level 2 evidence according to Scottish Intercollegiate Guidelines Network (SIGN) [111]. Pooled analyses or meta-analyses were excluded.

Most studies demonstrated inverse associations between the concentration of retinol and carotenoids and the risk of breast cancer in pre- or post-menopausal women [47,48,49,50,51,52,112,113,114,115,116], even though not all of them were statistically significant (Table 1) [52,113,114,116,117,118]. In particular, high serum or plasma concentrations of α- and β-carotene mostly exhibited a significantly reduced risk of invasive breast cancer, up to a 60–70% reduction in risk [47,48,49,50,51,52,112]. High levels of plasma carotenoids have also been found to be inversely associated with lower risks of overall cancer, as well as premalignant breast diseases such as benign breast disease or breast cancer in situ [113,118]. In addition, survival rates, recurrence risks, and other parameters of breast cancer were related with levels of retinol and carotenoids levels. A prospective study performed by Formelli et al. found that breast cancer patients with low plasma retinol levels showed lower overall survival than those with high retinol levels [119]. Carotenoids exhibited stronger inverse associations with the incidence, recurrence, and survival of aggressive breast cancer subtypes compared to recurrent/lethal types of breast cancer (β-carotene, RR = 0.74, 95% CI = 0.60–0.92, *P*-trend = 0.01, *P*-heterogeneity < 0.001) [50]. A clinical trial composing of 1551 women who had been previously treated for early stage breast cancer indicated that women with high plasma levels of total carotenoids had a significantly reduced risk of recurrent or new primary breast cancer [115]. Women with a higher risk of breast cancer, scored by genotype or mammographic density, exhibited a stronger inverse association between circulating carotenoid levels and breast cancer risk compared to women with lower risk of breast cancer [112]. More recent measurements of carotenoids before diagnosis showed a more apparent inverse association with breast cancer risk [50,52,116].

Despite the preventive roles of retinol and carotenoids proposed in several studies, some research observed minimal or no effects of retinol and carotenoids on breast cancer development [54,55,56,57,58]. Conflicting with previous results, a study (level 1 evidence) suggested that the use of vitamin A or carotenoids may increase the risk of breast cancer recurrence and death [120].

Breast cancer stratification based on estrogen receptor (ER), progesterone receptor (PR), and human epidermal growth factor receptor 2 status have become critical factors for predicting prognosis [15,70,121]. Wang et al. demonstrated that there was an inverse association between α-carotene and ER-positive breast cancer (OR = 0.63, 95% CI = 0.43–0.93, *P*-trend = 0.054), but not ER-negative breast cancer (OR = 0.86, 95% CI = 0.37–1.97, *P*-trend = 0.051) [48]. Bakker et al. reported that those with the highest quintile of α-carotene and β-carotene levels were 39–59% less likely to have ER-negative tumors (α-carotene, OR = 0.61, 95% CI = 0.39–0.98, *P*-trend = 0.02; β-carotene, OR = 0.41, 95% CI = 0.26–0.65, *P*-trend = 0.002), but no significant association was found in ER-positive tumors (α-carotene, OR = 0.77, 95% CI = 0.49–1.19, *P*-trend = 0.28; β-carotene, OR = 1.02, 95% CI = 0.66–1.57, *P*-trend = 0.91) [114]. Another analysis showed that those with the highest quintile of plasma carotenoids had 30–40% lower risks of both ER-positive and ER-negative breast cancer, but only a statistically significant risk reduction for ER-positive breast cancer [50]. Yan et al. found that serum α-carotene, β-carotene, lycopene, and lutein/zeaxanthin showed inverse associations with breast cancer risk regardless of hormone receptor status [47]. Experimental evidence from Prakash et al. showed that some carotenoids inhibited the cell growth of both ER-positive and ER-negative cells [122]. However, some data showed no differences on breast cancer risk according to ER status [112].

Tamimi et al. reported that higher concentrations of α-carotene and β-carotene were significantly associated with more than a 50% reduced risk of breast cancer with nodal metastases (α-carotene, OR = 0.39, 95% CI = 0.22–0.71, *P*-trend = 0.002; β-carotene, OR = 0.45, 95% CI = 0.24–0.82) [49]. However, Eliassen et al. demonstrated that there were no associations between carotenoids and the risk of breast cancer by tumor size, invasiveness, or nodal involvement [50].

Some observations suggested that the beneficial effects on the risk of breast cancer from carotenoids may be different depending on lifestyle factors associated with oxidative stress, such as smoking status or alcohol intake. Higher total carotenoid levels in plasma showed a reduced risk of breast cancer among smokers (smokers, OR = 0.55, 95% CI = 0.31–0.97, *P*-trend = 0.068; non-smokers, OR = 1.04, 95% CI = 0.63–1.72, *P*-trend = 0.52; *P*-interaction = 0.17) [48]. Epplein et al. also demonstrated that women who have ever smoked had a significant inverse association between total carotenoid levels and the risk of breast cancer (smokers, OR = 0.29, 95% CI = 0.10–0.85; non-smokers, OR = 1.19, 95% CI = 0.62–2.27, *P*-interaction = 0.17) [57]. A pooled analysis of eight cohort studies has revealed significantly stronger inverse associations between carotenoids and breast cancer risk among smokers [70]. Some explain the above results by suggesting that carotenoids counteract the ROS generated by smoke and inhibit smoke-stimulated insulin-like growth factor (IGF) signaling [123,124]. In contrast, Eliassen et al. confirmed that the association between α-carotene and breast cancer risk was significantly stronger in non-smokers compared to smokers (non-smokers, RR = 0.74, 95% CI = 0.60–0.92, *P*-trend = 0.01; smokers, RR = 1.23, 95% CI = 0.54–2.80, *P*-trend = 0.22; *P*-interaction = 0.03) [50]. There were no significant differences in breast cancer risks by alcohol consumption [50,70].

## 7. Vitamin A Actions through Nuclear Receptors and the Roles of Carotenoids in Regulation of Cell Signaling in Breast Cancer—Review of Preclinical and Clinical Studies

**Figure 3:**
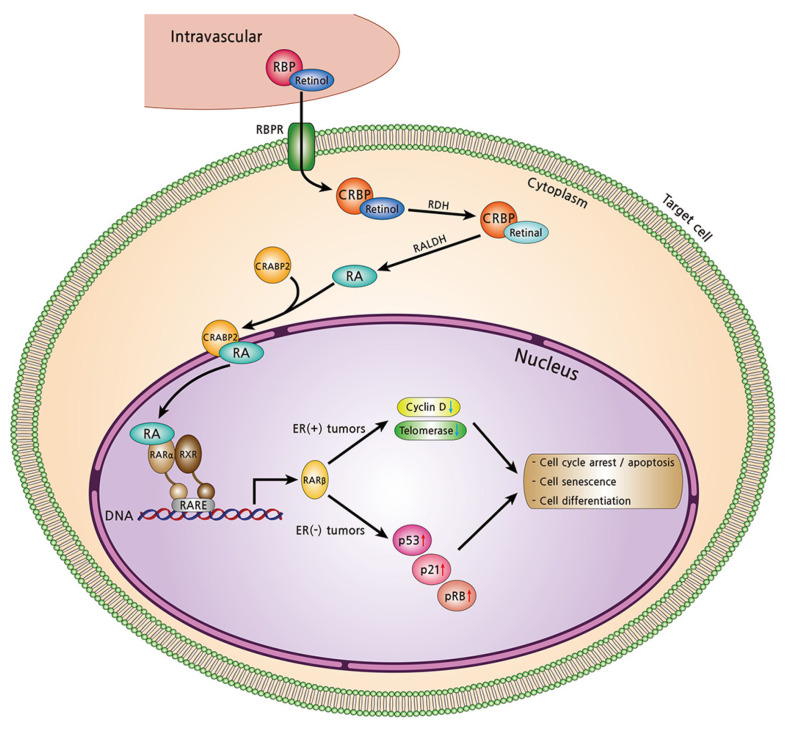
The cellular uptake of retinol by target cells (breast cancer cells) and its genetic regulation in the nucleus. Retinol circulates the bloodstream by binding to retinol binding protein (RBP). Retinol migrates into the target cell cytoplasm through RBP-receptor (RBPR). By binding to cellular RBPs (CRBPs), intracellular retinol is enzymatically converted into active forms. Through retinol dehydrogenase (RDH) reaction, retinol is converted to retinal. It is then converted to retinoic acid (RA) with three isomers (all-*trans*-, 9-*cis*-, and 13-*cis*-retinoic acid) by retinal dehydrogenase (RALDH). RA is delivered into the nucleus while bound to RARα by cellular retinoic acid-binding protein-2 (CRABP2). RA binds to dimers of retinoic acid receptor-α (RARα) and retinoid X receptors (RXRs) at retinoic acid response elements (RAREs), which are located in gene promoters. This complex promotes the expression of its downstream target genes, such as RARβ, inducing cell differentiation and growth inhibition. In ER-positive tumors, cell cycle arrest and senescence are caused by inhibition of cyclin D- and telomerase-related activities. In ER-negative tumors, stimulation of p53, p21, and retinoblastoma protein (pRB) leads to cell apoptosis and senescence.

The diverse functions of vitamin A are mediated by retinoic acid binding to retinoic acid receptors (RARs) and retinoid X receptors (RXRs), which are the steroid hormone nuclear receptors [125]. These activated receptors modulate the expression of genes encoding various structural proteins, binding proteins, and enzymes [126,127,128]. Changes in the expression levels of the RARs and RXRs are thought to be associated with retinoid-mediated carcinogenesis. Among three isotypes (α, β, and γ) of RARs and RXRs, RARβ and RXRβ were inactivated in various types of premalignant and malignant tissues, and these cancer cells had lower retinoid levels than normal cells [10,125]. A number of solid tumors, including breast cancer, showed reduced expression of RARβ2 mRNA levels. Indeed, methylation of the RARβ2 gene promoter was increased in patients with breast cancer [129,130]. Expression of the RARβ2 gene can induce apoptosis and growth arrest via retinoid-dependent and -independent pathways. As retinoids bind to RARα, the compound upregulates the RARβ gene, resulting in the stimulation of a number of cell differentiation and death genes and the inhibition of breast cancer metastasis in vivo (Figure 3) [131,132,133,134]. In one study, RARβ was induced in 33% of patients with breast cancer when treated with all-*trans*-retinoic acid for three weeks [135]. The findings that RARβ2 was activated by 9-*cis*- and 13-*cis*-retinoic acid treatment, and that increased RARβ2 levels were correlated with clinical response, suggested the tumor-suppressive effects of RARβ2 [10,136,137,138,139,140]. Breast cancer cell lines with no RARβ2 expression can achieve resistance to the growth-suppressive effect of retinoids [141].

According to the hormone receptor status of breast tumor cells, ER-positive breast cancer has been found to have relatively high levels of RARα and to be sensitive to retinoids, whereas ER-negative tumors have low levels of RARα and were found to be resistant to retinoids [131,142]. In ER-positive tumors, it is known that unliganded RARα paradoxically stimulates estrogen-dependent cell proliferation by interacting with ERα [143]. In contrast, retinoid-bound RARα cannot interact with ERα, resulting in anti-estrogenic activity. Although ER-negative tumors have little or no RARα, recent findings suggest that ER-negative tumors are considered to have significantly higher RARβ mRNA expression than ER-positive tumors. High RARβ expression can lead to acquired ATRA sensitivity, resulting in ATRA-dependent growth-inhibition [144]. An in vitro study suggested that retinoids inhibit tumor cell proliferation of both ER-positive and ER-negative breast cancer [122], but in different ways. In ER-positive tumors, retinoids inhibit the levels of cyclin D and telomerase, resulting in cell cycle arrest and senescence. In ER-negative tumors, retinoids stimulate the expression of p53, p21, and retinoblastoma protein leading to growth suppression [10,145]. However, a clinical trial comprised of patients with hormone-responsive metastatic breast cancer (level 1 evidence) showed no beneficial effects with the combination of hormonal therapy and retinoids [146]. Another clinical trial conducted in patients with metastatic breast cancer with ATRA plus paclitaxel treatment (level 2 evidence) showed 76.4% of clinical benefit [147].

**Figure 4:**
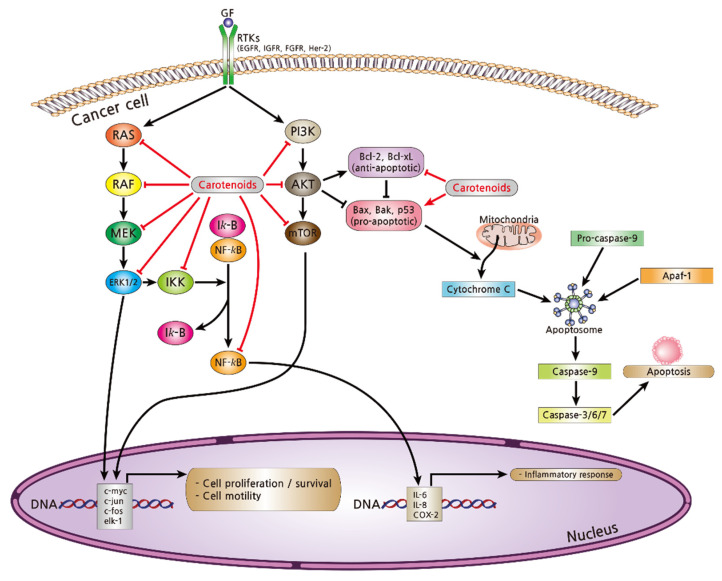
Breast oncogenesis is mediated by various signaling pathways. When growth factors bind to the receptor tyrosine kinases (RTKs; e.g., EGFR, IGFR, FGFR, and Her-2), the PI3K/Akt/mTOR pathway stimulates cell proliferation and increases cell motility. The Akt protein activates anti-apoptotic proteins, such as Bcl-2, Bcl-xL, and Bad, whereas it inhibits pro-apoptotic proteins, such as Bax, p53, therefore resulting in cell growth by inhibiting apoptosis. Another signaling pathway called the RAS/RAF/MEK/ERK1/2 pathway promotes the expression of transcription factors essential for cell survival and tumorigenesis, including c-Myc, c-Jun, c-Fos, and ELK-1. ERK1/2 activates Iκ-B kinase (IKK), inducing proteasomal degradation of Iκ-B, which is an inhibitory protein bound to NF-κB. The released NF-κB migrates into the nucleus and stimulates an inflammatory response by transcription of IL-6, IL-8, COX-2, and iNOS.

Breast cancer cells can achieve their ability to proliferate, survive, and invade as a result of the dysregulation of cellular signaling pathways (Figure 4) [15]. Among cancer signaling pathways, the phosphoinositide 3-kinase (PI3K)/protein kinase B (PKB or Akt)/mammalian target of rapamycin (mTOR) pathway promotes cellular proliferation and contributes to tumor occurrence and aggravation [148,149]. Activation of PI3K/Akt/mTOR signaling stimulates cell motility and initiates the metastatic phenotype of breast cancer cells [150]. Extracellular signaling-regulated kinase1/2 (ERK1/2) is a serine/threonine kinase that leads to tumor invasion [151]. Phosphorylation of ERK1/2 can activate nuclear factor-κB (NF-κB), a pro-inflammatory transcription factor, promoting the transcription of anti-apoptotic genes including Bcl-2 and B-cell lymphoma-extra-large (Bcl-xL) in the nucleus, thus inhibiting cell death and promoting survival [152,153,154]. In metastatic breast cancer, circulating tumor cells have significantly decreased apoptotic pathway signaling [155,156].

Carotenoids induce apoptosis in breast cancer cells by regulating these signaling pathways. Carotenoids downregulate the PI3K/Akt/mTOR pathway and inhibit RAS/RAF/MEK/ERK1/2 signaling, therefore inhibiting cell proliferation and motility. Carotenoids also inhibit the phosphorylation of IKK protein, thus blocking the degradation of Iκ-B. By inhibiting NF-κB activity and sequestering reactive oxygen species, pro-inflammatory mediators cannot be transcribed. Furthermore, carotenoids inhibit the activities of pro-survival proteins (Bcl-2 and Bcl-xL) and stimulate the expression of pro-death proteins (Bax, Bak, and p53). The activation of pro-death proteins promotes caspase activities leading to cancer cell death.

Numerous analyses have been performed to elucidate the mechanism for the protective effects of carotenoids on the tumorigenesis of breast cancer [157,158]. Carotenoids alleviate inflammation, which can reduce the alteration into cancer cells [159]. Astaxanthin reduces the growth of breast cancer cells by inhibiting the PI3K/Akt/mTOR pathway, followed by subsequently blocking translation of the MYC protein, which is essential in oncogenesis [160,161]. Similarly, lycopene halted the cell cycle progression phase and increased the expression of apoptosis-associated proteins [162,163,164]. Lycopene attenuated phosphorylation of Akt and mTOR in human breast cancer cell lines, enhancing the activation of pro-apoptotic Bcl-2-associated X protein (Bax) and p53 mRNA expression [165,166].

As well as lycopene, β-carotene also arrests the cell cycle and supports apoptosis [37]. An in vitro study suggested that β-carotene regulates the expression of genes that are sensitive to oxidative stress by inhibiting Akt and ERK1/2 signaling. β-carotene can suppress the expression of Bcl-2 and NF-κB and activate the family of cysteine-aspartic proteases called caspase 3, inducing apoptosis of breast cancer cells [167]. In addition, when fucoxanthin, a xanthophyll present in brown seaweeds, was pharmacologically administrated in a breast cancer cell line, it suppressed PI3K/Akt signaling and NF-κB levels in a concentration-dependent manner, leading to inhibition of the malignant phenotype [168]. Several investigations revealed ATRA and its derivative provoke breast cancer cell death by decreasing Bcl-2 activity and increasing Bax and caspase activities [169,170,171]. The carotenoids with anticancer drugs, such as doxorubicin, synergistically enhance apoptosis in breast cancer cells but not in normal cells [172].

Carotenoids perform anti-tumorigenic effects by several other mechanisms, including enhanced gap junction communication, stimulation of the antioxidant response element transcription system, inhibition of IGF-driven cell proliferation, or scavenging of ROS [70,173,174,175]. They also stimulate the immune system by increasing the levels of lymphocytes and natural killer cells [176].

## 8. The Potentials for Clinical Application of Vitamin A and Carotenoids as Innovative Therapeutic Agents against Breast Cancer

Efforts to utilize antioxidants such as vitamin A and carotenoids in breast cancer treatment have been made, as these compounds have cytotoxic effects on cancer cells without affecting normal cells, which can minimize the side effects of chemotherapeutic drugs [177]. Numerous studies demonstrated the synergistic effects of vitamin A and carotenoids with anticancer drugs in breast cancer, maximizing cell growth inhibition and apoptosis [178,179]. A recent study by Eid et al. demonstrated that fucoxanthin would synergistically interact with chemotherapeutic agents to overcome multidrug resistant breast cancer cells [180]. When carotenoids are combined with anti-cancer drugs, the activities of caspases and p53 were increased and the activities of metabolic enzymes were reduced. This enhanced the cytotoxicity of doxorubicin to cancer cells, while reducing the dose of the drug, thus overcoming the multidrug resistance of cancer cells.

Despite the protective roles of vitamin A and carotenoids in breast cancer development shown in preclinical and clinical research, its application in clinical practice is still limited due to the low solubility of lipophilic vitamin A and carotenoid compounds along with low bioavailability of carotenoid. A new strategy of encapsulating carotenoids with various nanocarriers has emerged to enhance drug delivery to cancer sites [181]. Several carotenoid-loaded nanotechnologies have been reported to have significant cytotoxic effects on breast cancer cells. Crocin, a carotenoid-derived natural compound that is responsible for the color of saffron, conjugated with synthesized gold nanoparticles (AuNPs), significantly decreased the growth of cancer cells [182]. The nanosized liposomal form of crocin also exhibited increased anti-tumor activity on breast cancer cells compared to crocin itself [183]. Leaky tumor microvasculature environments allow for nanoparticles to transport into cancer cells from blood vessels. On the other hand, the intact vasculature of other tissues keeps nanoparticles in the bloodstream. Another study by Huang et al. showed that the co-delivery of ATRA and paclitaxel using human serum albumin-based nanocarriers markedly reduced the metastatic properties of breast cancer cells both in vitro and in vivo compared to single drugs loaded with nanoparticles [184]. The conjugation of retinoids or carotenoids with nanotechnology not only improves the bioaccessibility of retinoids or carotenoids to its target cells, but also reduces cancer progression and metastasis, indicating it as a novel therapeutic strategy in cancer management.

## 9. Conclusions

Vitamin A and carotenoids are exceptionally efficient quencher of reactive oxygen species and responsible for protecting from photooxidative damage. The significance of vitamin A and carotenoids as powerful antioxidants against several cancers has been highlighted. In the past twenty years, considerable attempts have been made to reduce breast cancer risk by modifying lifestyle. In particular, the positive effects provided by vitamin A from high fruit and vegetable consumption have become widely studied. However, the clinical application of vitamin A and carotenoids in breast cancer treatment is limited due to inconsistencies among studies regarding the exact roles of vitamin A and carotenoids in breast cancer. This paper provides an updated comprehensive review of the functions and evaluation methods of vitamin A and carotenoids, along with their genetic variations associated with cancers and other diseases. The epidemiological evidence showed reduced breast cancer risk with at least one or more analytes of retinol and/or carotenoids through inhibition of cell proliferation, survival, and invasion, supporting the protective effects of vitamin A and carotenoids in breast cancer development and progression. Recent studies have encouraged the potential use of vitamin A and carotenoids as novel therapeutic agents by utilizing their anti-tumor and anti-metastatic properties without side effects through alternative delivery systems. These findings suggest the promising future of the clinical application of vitamin A and carotenoids in breast cancer prevention and treatment.

## Figures and Tables

**Table 1:** Prospective epidemiological studies of vitamin A and breast cancer risk, from 2000 to 2020.

Author, Publication Year	Study Design	# of Cases	# of Controls	Study Population ^1^	Follow-Up Period (Years)	Blood Measurement, Analysis Method	Quantity	Analytes	Risk Estimate (OR/RR/HR) (95% CI)	*P*-Trend	Outcome	*P*-Heterogeneity	Adjustment for Confounders
Peng et al., 2021 [112]	Nested case–control study	1919	1695	USA, 45–63 yrs, pre-m and post-m	≤20	Plasma, Reverse-phase HPLC	Quartile 4 vs. 1	Total carotenoids	RR = 0.80 (95% CI = 0.66–0.98)	0.006	BC risk	0.12	Age, BMI, FHx. of BC, Hx. of BBD, menopausal status, age at 1st birth, age at menarche, smoking, and alcohol
									RR = 0.73 (95% CI = 0.59–0.91)	0.008	ER+ BC risk		
									RR = 0.99 (95% CI = 0.67–1.47)	N.S.	ER– BC risk		
								α-carotene	RR = 0.80 (95% CI = 0.66–0.98)	0.03	BC risk	0.55	
									RR = 0.83 (95% CI = 0.67–1.03)	0.05	ER+ BC risk		
									RR = 0.70 (95% CI = 0.48–1.03)	0.05	ER– BC risk		
								β-carotene	RR = 0.82 (95% CI = 0.67–1.00)	0.01	BC risk	1.00	
									RR = 0.80 (95% CI = 0.64–1.00)	0.01	ER+ BC risk		
									RR = 0.78 (95% CI = 0.52–1.16)	N.S.	ER– BC risk		
								β-cryptoxanthin	RR = 0.89 (95% CI = 0.73–1.09)	N.S.	BC risk	0.21	
									RR = 0.88 (95% CI = 0.71–1.10)	N.S.	ER+ BC risk		
									RR = 1.07 (95% CI = 0.74–1.55)	N.S.	ER– BC risk		
								Lutein + zeaxanthin	RR = 0.83 (95% CI = 0.68–1.01)	0.01	BC risk	0.02	
									RR = 0.72 (95% CI = 0.58–0.89)	0.002	ER+ BC risk		
									RR = 1.28 (95% CI = 0.88–1.86)	N.S.	ER– BC risk		
								Lycopene	RR = 0.85 (95% CI = 0.70–1.03)	N.S.	BC risk	0.18	
									RR = 0.77 (95% CI = 0.63–0.95)	0.005	ER+ BC risk		
									RR = 0.98 (95% CI = 0.69–1.41)	N.S.	ER– BC risk		
Cohen et al., 2017 [113]	Nested case–control study	147 ^2^	293	Multiethnic, ≤50, pre-m and post-m	27.0 month	Plasma, HPLC	Tertile 3 vs. 1	Total carotenoids	OR = 0.83 (95% CI = 0.48–1.44)	N.S.	PBD risk		Age, BMI, FHx. of BC, and smoking
								α-carotene	OR = 0.68 (95% CI = 0.37–1.27)	N.S.	PBD risk		
								β-carotene	OR = 0.76 (95% CI = 0.43–1.34)	N.S.	PBD risk		
								β-cryptoxanthin	OR = 0.62 (95% CI = 0.36–1.09)	0.056	PBD risk		
								Lycopene	OR = 0.81 (95% CI = 0.49–1.35)	N.S.	PBD risk		
								Lutein + zeaxanthin	OR = 0.82 (95% CI = 0.47–1.43)	N.S.	PBD risk		
Bakker et al., 2016 [114]	Nested case–control study	1502	1502	Europe, 35–70 yrs, pre-m and post-m	≤13	Plasma, HPLC	Quintile 5 vs. 1	α-carotene	OR = 0.61 (95% CI = 0.39–0.98)	0.02	ER– BC risk	0.26	Age, menopausal status, menstrual phase, blood collection time, BMI, height, age at menarche, age at 1st birth, OC use, MHT, smoking, alcohol, education, and energy intakes
									OR = 0.77 (95% CI = 0.49–1.19)	N.S.	ER+ BC risk		
									OR = 0.64 (95% CI = 0.36–1.13)	N.S.	ER–/PR– BC risk	0.28	
								β-carotene	OR = 0.41 (95% CI = 0.26–0.65)	0.002	ER– BC risk	0.03	
									OR = 1.02 (95% CI = 0.66–1.57)	N.S.	ER+ BC risk		
									OR = 0.45 (95% CI = 0.26–0.80)	0.02	ER–/PR– BC risk	0.2	
								Lycopene	OR = 1.07 (95% CI = 0.56–2.03)	N.S.	ER– BC risk	0.26	
									OR = 0.90 (95% CI = 0.55–1.48)	N.S.	ER+ BC risk		
								Lutein	OR = 1.19 (95% CI = 0.66–2.13)	N.S.	ER– BC risk	0.11	
									OR = 0.59 (95% CI = 0.35–1.00)	N.S.	ER+ BC risk		
								Zeaxanthin	OR = 1.29 (95% CI = 0.69–2.42)	N.S.	ER– BC risk	0.33	
									OR = 0.84 (95% CI = 0.49–1.45)	N.S.	ER+ BC risk		
									OR = 2.34 (95% CI = 1.04–5.23)	N.S.	ER–/PR– BC risk	0.03	
								β-Cryptoxanthin	OR = 0.84 (95% CI = 0.51–1.37)	N.S.	ER– BC risk	0.66	
									OR = 0.70 (95% CI = 0.45–1.10)	N.S.	ER+ BC risk		
								Total carotenoids	OR = 0.64 (95% CI = 0.37–1.09)	N.S.	ER– BC risk	0.61	
									OR = 0.85 (95% CI = 0.53–1.37)	N.S.	ER+ BC risk		
								Retinol	OR = 1.65 (95% CI = 0.97–2.81)	N.S.	ER– BC risk	0.32	
									OR = 1.02 (95% CI = 0.64–1.63)	N.S.	ER+ BC risk		
									OR = 2.37 (95% CI = 1.20–4.67)	0.02	ER–/PR– BC risk	0.06	
Yan et al., 2016 [47]	Nested case–control study	521	521	China, 25–70 yrs, 674 pre-m and 368 post-m	N/A	Serum, HPLC	Quartile 4 vs. 1	α-carotene	OR = 0.44 (95% CI = 0.30–0.65)	<0.01	BC risk		BMI, residence, education, income, alcohol, and Hx. of BBD
									OR = 0.42 (95% CI = 0.21–0.81)	0.01	BC risk for post-m	0.13	
									OR = 0.43 (95% CI = 0.27–0.69)	<0.01	BC risk for pre-m		
									OR = 0.40 (95% CI = 0.21–0.74)	<0.01	ER– BC risk		
									OR = 0.44 (95% CI = 0.29–0.67)	<0.01	ER+ BC risk		
									OR = 0.35 (95% CI = 0.19–0.62)	<0.01	PR– BC risk		
									OR = 0.47 (95% CI = 0.31–0.73)	<0.01	PR+ BC risk		
								β-carotene	OR = 0.27 (95% CI = 0.18–0.40)	<0.01	BC risk		
									OR = 0.42 (95% CI = 0.22–0.74)	0.01	BC risk for post-m	0.05	
									OR = 0.24 (95% CI = 0.14–0.40)	<0.01	BC risk for pre-m		
									OR = 0.24 (95% CI = 0.12–0.45)	<0.01	ER– BC risk		
									OR = 0.27 (95% CI = 0.18–0.43)	<0.01	ER+ BC risk		
									OR = 0.25 (95% CI = 0.14–0.44)	<0.01	PR– BC risk		
									OR = 0.27 (95% CI = 0.17–0.43)	<0.01	PR+ BC risk		
								β-cryptoxanthin	OR = 0.71 (95% CI = 0.48–1.03)	N.S.	BC risk		
									OR = 0.70 (95% CI = 0.37–1.35)	N.S.	BC risk for post-m	0.83	
									OR = 0.65 (95% CI = 0.41–1.04)	N.S.	BC risk for pre-m		
									OR = 0.97 (95% CI = 0.52–1.80)	N.S.	ER– BC risk		
									OR = 0.58 (95% CI = 0.38–0.89)	<0.01	ER+ BC risk		
									OR = 0.77 (95% CI = 0.45–1.33)	N.S.	PR– BC risk		
									OR = 0.62 (95% CI = 0.41–0.94)	0.02	PR+ BC risk		
								Lycopene	OR = 0.41 (95% CI = 0.28–0.61)	<0.01	BC risk		
									OR = 0.56 (95% CI = 0.29–1.09)	N.S.	BC risk for post-m	0.01	
									OR = 0.36 (95% CI = 0.22–0.60)	<0.01	BC risk for pre-m		
									OR = 0.36 (95% CI = 0.19–0.68)	<0.01	ER– BC risk		
									OR = 0.39 (95% CI = 0.25–0.61)	<0.01	ER+ BC risk		
									OR = 0.28 (95% CI = 0.15–0.51)	<0.01	PR– BC risk		
									OR = 0.45 (95% CI = 0.28–0.70)	<0.01	PR+ BC risk		
								Lutein + zeaxanthin	OR = 0.26 (95% CI = 0.17–0.38)	<0.01	BC risk		
									OR = 0.28 (95% CI = 0.13–0.57)	<0.01	BC risk for post-m	0.21	
									OR = 0.25 (95% CI = 0.15–0.41)	<0.01	BC risk for pre-m		
									OR = 0.27 (95% CI = 0.14–0.52)	<0.01	ER– BC risk		
									OR = 0.25 (95% CI = 0.16–0.39)	<0.01	ER+ BC risk		
									OR = 0.24 (95% CI = 0.13–0.43)	<0.01	PR– BC risk		
									OR = 0.26 (95% CI = 0.17–0.42)	<0.01	PR+ BC risk		
Wang et al., 2015 [48]	Nested case–control study	496	496	USA, mean 69.4 yrs, post-m	≤9	Plasma, Reverse-phase HPLC	Quartile 4 vs. 1	α-carotene	OR = 0.50 (95% CI = 0.29–0.85)	0.041	BC risk		Age, Hx. of BBD, age at 1st birth, BMI, alcohol, smoking, and MHT
									OR = 0.63 (95% CI = 0.43–0.93)	0.054	ER+ BC risk	0.49	
									OR = 0.86 (95% CI = 0.37–1.97)	0.051	ER– BC risk		
								β-carotene	OR = 1.56 (95% CI = 0.90–2.72)	N.S.	BC risk		
								β-cryptoxanthin	OR = 1.01 (95% CI = 0.60–1.70)	N.S.	BC risk		
								Lycopene	OR = 0.95 (95% CI = 0.60–1.50)	N.S.	BC risk		
								Lutein + zeaxanthin	OR = 1.08 (95% CI = 0.65–1.80)	N.S.	BC risk		
								Total carotenoids	OR = 0.86 (95% CI = 0.56–1.33)	N.S.	BC risk		
Eliassen et al., 2015 [50]	Nested case–control study	2188 (1750 invasive BC)	2188	USA, 43–80 yrs, pre-m and post-m	≤20 (median 9.3)	Plasma, Reverse-phase HPLC	Quintile 5 vs. 1	α-carotene	RR = 0.74 (95% CI = 0.60–0.91)	0.01	BC risk		BMI, weight gain, ages at menarche, 1st birth, menopausal status, parity, alcohol, Hx. Of BBD, and FHx. of BC
									RR = 0.74 (95% CI = 0.60–0.92)	0.01	BC risk for nonsmokers	0.03	
									RR = 0.74 (95% CI = 0.59–0.93)	0.02	ER+ BC risk	0.94	
									RR = 0.54 (95% CI = 0.35–0.83)	0.01	Risk of BC recurrence/death	0.08	
								β-carotene	RR = 0.72 (95% CI = 0.59–0.88)	<0.001	BC risk		
									RR = 0.73 (95% CI = 0.59–0.91)	0.002	BC risk for nonsmokers	0.23	
									RR = 0.70 (95% CI = 0.56–0.89)	0.002	ER+ BC risk	0.28	
									RR = 0.32 (95% CI = 0.21–0.51)	<0.001	Risk of BC recurrence/death	<0.001	
								Total carotenoids	RR = 0.77 (95% CI = 0.63–0.94)	0.005	BC risk		
									RR = 0.82 (95% CI = 0.66–1.01)	0.04	BC risk for nonsmokers	0.45	
									RR = 0.73 (95% CI = 0.58–0.92)	0.003	ER+ BC risk	0.2	
									RR = 0.48 (95% CI = 0.31–0.73)	0.001	Risk of BC recurrence/death	0.02	
								Lycopene	RR = 0.82 (95% CI = 0.67–1.01)	0.02	BC risk		
								β-cryptoxanthin	RR = 0.86 (95% CI = 0.70–1.06)	N.S.	BC risk		
								Lutein + zeaxanthin	RR = 0.86 (95% CI = 0.70–1.05)	N.S.	BC risk		
Sisti et al., 2015 [117]	Nested case–control study	1179 (535 pre-m at Dx. and 492 post-m at Dx.)	1179	USA, average 46 yrs, pre-m	≤34	Plasma, HPLC	Quartile 4 vs. 1	α-carotene	RR = 1.17 (95% CI = 0.81–1.68)	N.S.	BC risk for pre-m at Dx.	0.05	BMI, age at menarche, age at 1st birth and parity, FHx. of BC, Hx. of BBD, and alcohol
									RR = 0.73 (95% CI = 0.49–1.06)	N.S.	BC risk for post-m at Dx.		
								β-carotene	RR = 0.98 (95% CI = 0.68–1.41)	N.S.	BC risk for pre-m at Dx.	0.41	
									RR = 0.95 (95% CI = 0.63–1.42)	N.S.	BC risk for post-m at Dx.		
								β-cryptoxanthin	RR = 0.87 (95% CI = 0.59–1.27)	N.S.	BC risk for pre-m at Dx.	0.4	
									RR = 0.85 (95% CI = 0.57–1.26)	N.S.	BC risk for post-m at Dx.		
								Lutein + zeathanthin	RR = 1.19 (95% CI = 0.81–1.54)	N.S.	BC risk for pre-m at Dx.	0.13	
									RR = 0.89 (95% CI = 0.60–1.33)	N.S.	BC risk for post-m at Dx.		
								Lycopene	RR = 1.00 (95% CI = 0.70–1.42)	N.S.	BC risk for pre-m at Dx.	0.03	
									RR = 0.66 (95% CI = 0.45–0.96)	0.02	BC risk for post-m at Dx.		
								Total carotenoids	RR = 1.13 (95% CI = 0.78–1.62)	N.S.	BC risk for pre-m at Dx.	0.04	
									RR = 0.79 (95% CI = 0.53–1.19)	N.S.	BC risk for post-m at Dx.		
Pouchieu et al., 2014 [118]	Nested case–control study	159 cancer cases ^3^ (100 BC cases)	159 controls (100 BC controls)	France, mean 51.6 yrs, 43 pre-m and 57 post-m for BC cases	7.5	Plasma, HPLC	OR for an increase of 0.1 μmol/L	β-carotene	OR = 0.95 (95% CI = 0.90–0.99)	0.04	Overall cancer risk		Age, intervention group, number of dietary records, BMI, height, smoking, alcohol, physical activity, education, FHx. of BC, menopausal status, OC use, number of parity, and energy/lipid/fruit/vegetable intakes
									OR = 0.96 (95% CI = 0.89–1.03)	N.S.	BC risk		
								α-carotene	OR = 0.93 (95% CI = 0.79–1.09)	N.S.	Overall cancer risk		
									OR = 0.94 (95% CI = 0.77–1.15)	N.S.	BC risk		
								β-cryptoxanthin	OR = 0.89 (95% CI = 0.81–0.99)	0.03	Overall cancer risk		
									OR = 0.83 (95% CI = 0.71–0.96)	0.02	BC risk		
								Lycopene	OR = 1.07 (95% CI = 0.99–1.15)	N.S.	Overall cancer risk		
									OR = 1.04 (95% CI = 0.95–1.13)	N.S.	BC risk		
								Lutein	OR = 1.06 (95% CI = 0.85–1.31)	N.S.	Overall cancer risk		
									OR = 1.06 (95% CI = 0.72–1.56)	N.S.	BC risk		
								Retinol	OR = 1.00 (95% CI = 0.95–1.06)	N.S.	Overall cancer risk		
									OR = 0.99 (95% CI = 0.91–1.09)	N.S.	BC risk		
Maillard et al., 2010 [56]	Nested case–control study	366 (84 pre-m and 282 post-m)	720	France, 40–65 yrs, pre-m and post-m	7	Serum, HPLC	Quintile 5 vs. 1	Total carotenoids	OR = 0.74 (95% CI = 0.47–1.16)	N.S.	BC risk		Age, menopausal status, fasting status, time of blood collection, alcohol, height, MHT, education, age at 1st birth and parity, FHx. of BC, and Hx. of BBD
								Retinol	OR = 0.85 (95% CI = 0.53–1.35)	N.S.	BC risk		
								Lutein	OR = 0.97 (95% CI = 0.63–1.50)	N.S.	BC risk		
								Zeaxanthin	OR = 0.77 (95% CI = 0.48–1.23)	N.S.	BC risk		
								β-cryptoxanthin	OR = 1.02 (95% CI = 0.65–1.60)	N.S.	BC risk		
								Canthaxanthin	OR = 1.02 (95% CI = 0.66–1.60)	N.S.	BC risk		
								α-carotene	OR = 0.99 (95% CI = 0.62–1.56)	N.S.	BC risk		
								β-carotene	OR = 0.85 (95% CI = 0.53–1.35)	N.S.	BC risk		
								Lycopene	OR = 0.95 (95% CI = 0.58–1.55)	N.S.	BC risk		
Dorjgochoo et al., 2009 [55]	Nested case–control study	365	726	China, 40–70 yrs, 542 pre-m and 549 post-m	7.5	Plasma, Reverse-phase HPLC	Quartile 4 vs. 1	Retinol	OR = 1.17 (95% CI = 0.77–1.78)	N.S.	BC risk		Age, education, occupation, age at menarche, age at 1st birth, W:H, exercise, smoking, menopausal status, Hx. of BBD, FHx. of BC, energy/furit/vegetable intakes, batch for assays, and other plasma lipophilic antioxidants
									OR = 1.30 (95% CI = 0.68–2.48)	N.S.	BC risk for pre-m		
									OR = 1.05 (95% CI = 0.56–1.99)	N.S.	BC risk for post-m		
								Total carotenoids	OR = 1.30 (95% CI = 0.87–1.93)	N.S.	BC risk		
									OR = 1.06 (95% CI = 0.51–1.91)	N.S.	BC risk for pre-m		
									OR = 1.77 (95% CI = 0.96–3.25)	0.05	BC risk for post-m		
								β-carotene (*trans*- + *cis*-)	OR = 1.47 (95% CI = 0.92–2.35)	N.S.	BC risk		
									OR = 1.44 (95% CI = 0.73–2.82)	N.S.	BC risk for pre-m		
									OR = 1.58 (95% CI = 0.78–3.19)	N.S.	BC risk for post-m		
								α-carotene (*trans*-)	OR = 0.98 (95% CI = 0.62–1.54)	N.S.	BC risk		
								Lycopene	OR = 0.83 (95% CI = 0.49–1.39)	N.S.	BC risk		
									OR = 0.66 (95% CI = 0.30–1.43)	N.S.	BC risk for pre-m		
									OR = 1.17 (95% CI = 0.54–2.51)	N.S.	BC risk for post-m		
								Lutein + zeaxanthin (*trans*-)	OR = 1.02 (95% CI = 0.67–1.54)	N.S.	BC risk		
								Lutein + zeaxanthin (*cis*-)	OR = 1.10 (95% CI = 0.65–1.85)	N.S.	BC risk		
								β-cryptoxanthin (*trans*-)	OR = 1.25 (95% CI = 0.75–2.09)	N.S.	BC risk		
									OR = 1.36 (95% CI = 0.63–2.94)	N.S.	BC risk for pre-m		
									OR = 1.21 (95% CI = 0.56–2.59)	N.S.	BC risk for post-m		
								β-cryptoxanthin (*cis*-)	OR = 0.94 (95% CI = 0.53–1.67)	N.S.	BC risk		
Epplein et al., 2009 [57]	Multiethnic Cohort Study	286	535	Multiethnic, 45–75 yrs, post-m	≤2.5 (median 1 year and 5 months)	Plasma, HPLC	Quartile 4 vs. 1	α-carotene	OR = 0.88 (95% CI = 0.56–1.39)	N.S.	BC risk		Year of 1st birth, geographic area, ethnicity, time of blood collection, fasting status, MHT, age, BMI, alcohol, age at menarche/menopause/1st birth, and number of full-term pregnancies
								β-carotene	OR = 0.73 (95% CI = 0.46–1.15)	N.S.	BC risk		
								β-cryptoxanthin (*cis*-)	OR = 1.27 (95% CI = 0.81–1.99)	N.S.	BC risk		
								Lycopene	OR = 0.88 (95% CI = 0.57–1.38)	N.S.	BC risk		
								Total carotenoids	OR = 0.80 (95% CI = 0.51–1.26)	N.S.	BC risk		
									OR = 0.29 (95% CI = 0.10–0.85)	N/A	BC risk for ever smokers (data not shown)	0.17	
								Lutein + zeaxanthin (*cis*-)	OR = 0.75 (95% CI = 0.48–1.16)	N.S.	BC risk		
								Retinol	OR = 1.13 (95% CI = 0.73–1.76)	N.S.	BC risk		
Kabat et al., 2009 [52]	Prospective cohort study	190 (153 invasive and 37 in situ)	5260 non-cases	USA,50–79 yrs, 5450 post-m	8	Serum, Reverse-phase HPLC	Tertile 3 vs. 1	Retinol	HR = 0.96 (95% CI = 0.62–1.49)	N.S.	BC risk		Age, education, ethnicity, BMI, OC use, MHT, age at menarche/1st birth/menopause, alcohol, FHx. of BC, physical activity, randomization status, and micronutrient/energy intakes
								α-carotene	HR = 0.55 (95% CI = 0.34–0.90)	0.02	BC risk		
									HR = 0.42 (95% CI = 0.23–0.75)	0.002	BC risk of conc. measured 1–3 yrs before Dx.		
								β-carotene	HR = 0.78 (95% CI = 0.49–1.24)	N.S.	BC risk		
									HR = 0.34 (95% CI = 0.19–0.61)	0.0002	BC risk of conc. measured 1–3 yrs before Dx.		
								β-cryptoxanthin	HR = 1.14 (95% CI = 0.73–1.79)	N.S.	BC risk		
								Lycopene	HR = 1.47 (95% CI = 0.98–2.22)	N.S.	BC risk		
								Lutein + zeaxanthin	HR = 0.91 (95% CI = 0.59–1.38)	N.S.	BC risk		
Sesso et al., 2005 [54]	Nested case–control study	508 (344 ER+/PR+ BC)	508	USA, ≥45 yrs (mean 54 yrs), pre-m and post-m	7	Plasma, Reverse-phase HPLC	Quartile 4 vs. 1	α-carotene	RR = 1.06 (95% CI = 0.61–1.84)	N.S.	BC risk		Age, smoking, aspirin/vitamin Tx., plasma cholesterol, BMI, FHx. of BC, physical activity, age at menarche/1st pregnancy, OC use, number of pregnancies, monopausal status, MHT, alcohol, and fat/fiber/fruit/vegetable intakes
								β-carotene	RR = 1.36 (95% CI = 0.79–2.33)	N.S.	BC risk		
								Lycopene	RR = 0.93 (95% CI = 0.56–1.52)	N.S.	BC risk		
									RR = 0.90 (95% CI = 0.47–1.71)	N.S.	ER+/PR+ BC risk	N/A	
								β-cryptoxanthin	RR = 0.82 (95% CI = 0.46–1.44)	N.S.	BC risk		
								Lutein + zeaxanthin	RR = 0.78 (95% CI = 0.45–1.38)	N.S.	BC risk		
Rock et al., 2005 [115]	Prospective cohort study ^4^	1551 previously treated for BC (205 recurrent or new primary BC)	N/A	USA, up to 4 yrs post-diagnosis and completed initial Tx. (e.g., surgery, CTx., RTx.)	86 months	Plasma, HPLC	Quartile 4 vs. 1	Total carotenoids (α-carotene + β-carotene + Lutein + Lycopene + β-cryptoxanthin)	HR = 0.57 (95% CI = 0.37–0.88)	N/A	BC recurrence risk		Age at Dx., plasma cholesterol, BMI, tumor hormone receptors, and adjuvant CTx
Tamimi et al., 2005 [49]	Nested case–control study	969 (206 pre-m and 666 post-m)	969	USA, 43–70 yrs (mean 57 yrs), 418 pre-m and 1329 post-m	≤9 (median 4)	Plasma, HPLC	Quintile 5 vs1	α-carotene	OR = 0.64 (95% CI = 0.47–0.88)	0.01	BC risk		Age, menopausal status, MHT (use/duration), time of blood collection, fasting status, Age at menopause/menarche/1st birth, parity, BMI, weight gain, Hx. of BBD, FHx. of BC, and alcohol
									OR = 0.39 (95% CI = 0.22–0.71)	0.002	risk of BC with nodal metastasis	0.02	
									OR = 0.50 (95% CI = 0.28–0.91)	0.05	ER– BC risk	0.48	
									OR = 0.72 (95% CI = 0.50–1.04)	0.03	ER+ BC risk		
									OR = 0.40 (95% CI = 0.21–0.76)	0.02	risk of BC with poor differentiation	0.03	
								β-carotene	OR = 0.73 (95% CI = 0.53–1.02)	0.01	BC risk		
									OR = 0.45 (95% CI = 0.24–0.82)	N/A	risk of BC with nodal metastasis (data not shown)	N/A	
								β-cryptoxanthin	OR = 0.95 (95% CI = 0.69–1.31)	N.S.	BC risk		
								Lutein + zeaxanthin	OR = 0.74 (95% CI = 0.55–1.01)	0.04	BC risk		
								Lycopene	OR = 1.01 (95% CI = 0.73–1.39)	N.S.	BC risk		
								Total carotenoids	OR = 0.76 (95% CI = 0.55–1.05)	0.05	BC risk		
								Retinol	OR = 0.78 (95% CI = 0.56–1.07)	N.S.	BC risk		
Sato et al., 2002 [116] (1974 cohort)	Prospective cohort study	244	244	USA, mean 51.3 yrs (cases) and 51.1 yrs (controls), pre-m and post-m	≤20	Serum, HPLC	Quintile 5 vs. 1	Retinol	OR = 0.97 (95% CI = 0.53–1.80)	N.S.	BC risk		Age, race, menopausal status, time of blood collection, FHx. of BC, age at 1st birth, age at menarche, alcohol, smoking, BMI, duration of lactation, education, time since last meal, and total cholesterol
								α-carotene	OR = 0.69 (95% CI = 0.36–1.34)	N.S.	BC risk		
								β-carotene	OR = 0.41 (95% CI = 0.22–0.79)	0.007	BC risk		
									OR = 0.37 (95% CI = 0.15–0.93) ^5^	0.05	BC risk of conc. measured 10–15 yrs before Dx.		
								β-cryptoxanthin	OR = 0.98 (95% CI = 0.55–1.75)	N.S.	BC risk		
								Lutein	OR = 0.77 (95% CI = 0.43–1.40)	N.S.	BC risk		
								Lycopene	OR = 0.55 (95% CI = 0.29–1.06)	0.04	BC risk		
									OR = 0.49 (95% CI = 0.20–1.20)^5^	N.S.	BC risk of conc. measured 10–15 yrs before Dx.		
								Total carotenoids	OR = 0.55 (95% CI = 0.29–1.03)	0.02	BC risk		
Sato et al., 2002 [116] (1989 cohort)	Prospective cohort study	115	115	USA, mean 60.4 yrs (cases) and 60.2 yrs (controls), pre-m and post-m	≤5	Plasma, HPLC	Quintile 5 vs. 1	Retinol	OR = 1.03 (95% CI = 0.40–2.64)	N.S.	BC risk		Age, race, menopausal status, time of blood collection, FHx. of BC, age at 1st birth, age at menarche, alcohol, smoking, BMI, duration of lactation, education, time since last meal, and total cholesterol
								α-carotene	OR = 0.84 (95% CI = 0.34–2.08)	N.S.	BC risk		
								β-carotene	OR = 0.62 (95% CI = 0.27–1.42)	N.S.	BC risk		
								β-cryptoxanthin	OR = 0.70 (95% CI = 0.29–1.73)	N.S.	BC risk		
								Lutein	OR = 0.40 (95% CI = 0.17–0.98)	N.S.	BC risk		
								Lycopene	OR = 0.80 (95% CI = 0.34–1.85)	N.S.	BC risk		
								Total carotenoids	OR = 0.61 (95% CI = 0.26–1.43)	N.S.	BC risk		
Toniolo et al., 2001 [51]	Nested case–control study	270	270	USA, 35–65 yrs, 125 pre-m and 145 post-m (each)	≤9	Serum, HPLC	Quartile 1 vs. 4	Lutein	OR = 2.08 (95% CI = 1.11–3.90)	0.01	BC risk		Age, age at 1st birth, FHx. of BC, Hx. of BBD, and total cholesterol
								Zeaxanthin	OR = 1.12 (95% CI = 0.59–2.13)	N.S.	BC risk		
								β-cryptoxanthin	OR = 1.68 (95% CI = 0.99–2.86)	0.05	BC risk		
								Lycopene	OR = 1.50 (95% CI = 0.88–2.54)	N.S.	BC risk		
								α-carotene	OR = 1.99 (95% CI = 1.18–3.34)	0.0006	BC risk		
								β-carotene	OR = 2.21 (95% CI = 1.29–3.79)	0.006	BC risk		
								Total carotenoids	OR = 2.31 (95% CI = 0.35–3.96)	0.0008	BC risk		
								Retinol	OR = 0.78 (95% CI = 0.45–1.35)	N.S.	BC risk		
Hultén et al., 2001 [58](VIP + MONICA cohorts)	Prospective cohort study	124	246	Sweden, mean 52 yrs, pre-m and post-m	13 for VIP, 10 for MONICA	Plasma, HPLC	Quartile 4 vs. 1	α-carotene	RR = 0.70 (95% CI = 0.20–2.40)	N.S.	BC risk for pre-m		
									RR = 0.50 (95% CI = 0.20–1.40)	N.S.	BC risk for post-m		Age, BMI, total cholesterol, and triglycerides
								β-carotene	RR = 1.60 (95% CI = 0.50–5.40)	N.S.	BC risk for pre-m		
									RR = 0.70 (95% CI = 0.20–1.90)	N.S.	BC risk for post-m		
								β-cryptoxanthin	RR = 1.00 (95% CI = 0.30–3.60)	N.S.	BC risk for pre-m		
									RR = 0.80 (95% CI = 0.30–2.30)	N.S.	BC risk for post-m		
								Lycopene	RR = 1.20 (95% CI = 0.30–4.80)	N.S.	BC risk for pre-m		
									RR = 2.40 (95% CI = 0.70–7.90)	N.S.	BC risk for post-m		
								Lutein	RR = 0.30 (95% CI = 0.10–1.40)	0.03	BC risk for pre-m		
									RR = 0.90 (95% CI = 0.30–2.60)	N.S.	BC risk for post-m		
								Zeaxanthin	RR = 0.70 (95% CI = 0.20–3.30)	N.S.	BC risk for pre-m		
									RR = 0.40 (95% CI = 0.10–1.40)	N.S.	BC risk for post-m		
								Retinol	RR = 0.80 (95% CI = 0.20–3.30)	N.S.	BC risk for pre-m		
									RR = 0.60 (95% CI = 0.20–1.20)	N.S.	BC risk for post-m		
Hultén et al., 2001 [58] (MSP cohort)	Prospective cohort study	77	144	Sweden, mean 59 yrs, post-m	3	Plasma, HPLC	Quartile 4 vs. 1	α-carotene	RR = 0.60 (95% CI = 0.20–1.60)	N.S.	BC risk		Age, BMI, total cholesterol, and triglycerides
								β-carotene	RR = 0.40 (95% CI = 0.10–1.20)	N.S.	BC risk		
								β-cryptoxanthin	RR = 0.70 (95% CI = 0.20–2.00)	N.S.	BC risk		
								Lycopene	RR = 0.20 (95% CI = 0.00–0.70)	0.01	BC risk		
								Lutein	RR = 1.20 (95% CI = 0.40–3.70)	N.S.	BC risk		
								Zeaxanthin	RR = 1.30 (95% CI = 0.50–3.50)	N.S.	BC risk		
								Retinol	RR = 1.50 (95% CI = 0.50–4.60)	N.S.	BC risk		

^1^ Nationality, age, menopausal status. ^2^ Benign breast disease or breast cancer in situ. ^3^ 100 breast cancers, 29 prostate, 23 colorectal, 8 lung, and 9 upper respiratory tract cancers. ^4^ Study with level 1 evidence. ^5^ Tertile 3 vs. 1. Risk estimate (OR, RR, HR) above 1 indicated increased risk. Risk estimate below 1 indicates decreased risk. Abbreviations: OR, odds ratio; RR, relative risk; HR, hazard ratio; 95% CI, 95% confidence interval; yrs, years; pre-m, pre-menopausal women; post-m, post-menopausal women; HPLC, high pressure liquid chromatography; N.S., not significant; BC, invasive breast cancer; ER, estrogen receptor; PR, progesteron receptor; +, positive; –, negative; FHx., family history; Hx., history; BBD, benign breast disease; BMI, body mass index; OC, oral contraceptive; MHT, menopausal hormone therapy; W:H, waist to hips ratio; conc., concentrations; Tx., treatment; CTx., chemotherapy; RTx., radiotherapy; PBD, premalignant breast disease including benign breast disease or breast cancer in situ; and N/A, not applicable.
